# Hepatitis B virus-associated intrahepatic cholangiocarcinoma: a malignancy of distinctive characteristics between hepatocellular carcinoma and intrahepatic cholangiocarcinoma

**DOI:** 10.18632/oncotarget.14079

**Published:** 2016-12-21

**Authors:** Seogsong Jeong, Ying Tong, Meng Sha, Jinyang Gu, Qiang Xia

**Affiliations:** ^1^ Department of Liver Surgery, Renji Hospital, School of Medicine, Shanghai Jiaotong University, Shanghai, China

**Keywords:** hepatitis B virus-associated intrahepatic cholangiocarcinoma, prognosis, treatments, cancer-associated lymphangiogenesis, mesenchymal-to-lymphatic endothelial transition

## Abstract

It has been a decade since hepatitis B virus infection was identified as an etiological factor for the development of intrahepatic cholangiocarcinoma (ICC). In recent years, several studies have elucidated the critical impact of hepatitis B virus in ICC that significantly influenced the clinicopathological characteristics of ICC patients with intrahepatic cholangiocarcinoma. Distinctive features of patients with hepatitis B virus-associated ICC included younger age, preponderance of male patients, frequent elevation of alpha-fetoprotein, and infrequent lymph node metastasis. Furthermore, several studies indicated that the presence of hepatitis B virus is a favorable prognostic factor in terms of overall survival and relapse-free survival. However, there are also a few studies demonstrating that hepatitis B virus negatively influenced or showed no significant association with survival outcomes of patients with ICC. At present, there are no consensus on diagnostic procedures and treatments for such population. Therefore, we elucidated current knowledge and recent identifications of HBV-associated ICC to clarify the impact of chronic HBV infection on patients with ICC and to precisely conduct diagnostic procedures and curative treatments for HBV-associated ICC.

Intrahepatic cholangiocarcinoma (ICC) is a poorly understood, aggressive malignancy with an increasing incidence, and etiological factors are just starting to be elucidated [1, 2]. Previous publications on this malignancy are severely limited principally to clinical series from specialized hepatobiliary surgery. In recent years, several studies indicated that hepatitis B virus (HBV) infection, which is a common cause of both liver cirrhosis and hepatocelluar carcinoma (HCC), is also found to be a risk factor for ICC. HBV-associated ICC patients displayed significantly different clinicopathological characteristics as well as survival outcomes [3–5]. However, there are no diagnostic procedures and treatment prescription for this group of patients. In addition, many aspects of this relatively rare malignant disease are insufficiently understood, rendering the present literature hard to interpret. HBV-associated ICC lacks evaluation and its diagnosis and treatment have been the topic of few studies. In this review, we report current knowledge and recent discoveries on HBV-associated ICC so that clinicians need to be aware of establishing curative treatments efficiently.

## HBV INFECTION AND THE DEVELOPMENT OF ICC

To date, several risk factors for ICC have been identified, such as primary sclerosing cholangitis (PSC), hepatolithiasis, Caroli's disease, parasite infestation, hepatitis virus infection, and the risk factors vary according to region [6, 7]. Recently, hepatitis B virus has been associated with an increasing incidence of ICC in East Asian countries where it is endemic [8, 9]. A Japanese experience indicated that a seroprevalence of hepatitis B surface antigen (HBsAg) and hepatitis C virus (HCV) was found in 10% and 20% of patients with ICC, respectively [10]. In addition, Li et al. [11] used a diagnostic kit to detect the presence of serum HBV DNA in 104 of 183 (56.83%) sporadic ICC patients, confirming that seropositivity for HBsAg is indeed found to be a risk factor for ICC. Another study confirmed the generation of HBx protein in 70.4% (38/54) of paraffin-embedded HBV-associated ICC specimens, indicating that HBx may play a significant role in the development of ICC [12]. Furthermore, a meta-analysis demonstrated that all included studies revealed a statistically significant increased risk for ICC among ICC patients with HBV infection (rate ratio [RR], 2.66; 95% confidence interval [CI], 1.97 to 3.60), and the pooled risk estimate of Asian publications (RR, 3.63; 95% CI, 2.56 to 5.13) was relatively higher than studies from other regions (RR, 1.93; 95% CI, 0.78 to 4.76) [13]. However, there are also a few publications which could not confirm a significant association between viral hepatitis and the incidence of ICC [14]. In summary, there is consistent epidemiological evidence supporting a correlation between HBV infection and ICC, especially where HBV is endemic. However, current information is not enough to elucidate a specific mechanism to support the link between HBV and ICC.

In previous relevant publications, the distribution of HBV-associated ICC was found to vary worldwide with the lowest distribution found in US (1/625, 0.2%; 1/83, 1.2%) and the highest in China (154/317, 48.6%; 31/98, 31.6%; 26/97, 26.8%) [25–28]. The other Asian countries such as Korea (4/51, 7.8%; 84/622, 13.5%; 37/292, 12.7%) and Japan (2/50, 4%; 3/41, 7.3%) were found to have a moderate distribution of HBV-associated ICC [5, 8, 29–31].

## CLASSIFICATION

A consensus on the classification of ICC has yet to be reached due to inconsistencies in definition. In 2003, Yamasaki et al. [33] demonstrated that ICC can be stratified into three different subtypes: intraductal, periductal infiltrating, and mass-forming according to its morphological features. Previous studies revealed that intraductal and periductal types develop from the malignant transformation of epithelial cells of relatively large bile ducts, whereas mass-forming type originates from bi-potential hepatic stem cells inner portal areas or relatively small bile ducts [34]. A few etiological factors of ICC, such as hepatolithiasis and *Clonorchis sinensis* infection, were found to be associated with the intraductal type [35–37]. In contrast, most ICC patients with HBV infection were found to have the mass-forming type [38, 39]. On the basis of the histological features, ICC could also be further stratified into two different types: cholangiolar type and bile duct type. According to a previous publication, Yu et al. [40] suggested that the presence of viral hepatitis infection, including HBV and HCV, was found to be associated with the cholangiolar type of ICC (OR = 2.71; P = 0.008).

## CLINICOPATHOLOGICAL CHARACTERISTICS

To date, only a few recent studies have elucidated the role of HBV infection in the development of ICC and demonstrated that the clinicopathological features of HBV-associated ICC were significantly different from patients without HBV infection. Zhou et al. [42] by comparing the clinicopathological characteristics of 154 HBsAg (hepatitis B surface antigen)-positive ICC patients and 163 seronegative-HBsAg ICC patients discovered that HBsAg-positive ICC patients were found to be younger and had a higher male distribution, a higher incidence of cirrhosis and tumor encapsulation, frequent microvascular invasion, poorer tumor differentiation, a lower distribution of abnormal serum carbohydrate antigen 19-9 (CA19-9) level, and infrequent lymph node metastasis with abnormal aminotransferase levels, elevated serum alpha-fetoprotein (AFP) levels. In addition, the age and gender distribution was almost equal between HBsAg-positive ICC and HBV-associated HCC. Research of Zhou et al. [42] and Peng et al. [27] further indicated that neutrophilic infiltration was found to be more common in HBV-associated ICC patients. More recently, Tao et al. [28] found that prothrombin time was also found to be higher in HBV-associated ICC patients (P = 0.030), complementing the studies from Zhou et al. [42] and Peng et al [27]. The above mentioned clinicopathological characteristics of HBV-associated ICC were verified by a meta-analysis that involved 14 studies and 2842 cases [43]. HBV infection was more frequent in male patients [odds ratio (OR) = 1.91, 95% confidence interval (CI) = 1.06 to 3.44] and was correlated with elevated serum aspartate transaminase (AST) (OR = 1.93, 95%CI = 1.11 to 3.35) and AFP levels (OR = 3.86, 95%CI = 2.58 to 5.78), as well as a relatively lower level of serum CA19-9 (OR = 0.47, 95%CI = 0.34 to 0.65) [43]. In addition, there were significant differences in the incidence of liver cirrhosis (OR = 6.44, 95%CI = 4.33 to 9.56), capsule formation (OR = 6.04, 95%CI = 3.56 to 10.26), and lymph node metastasis (OR = 0.39, 95%CI = 0.25 to 0.58).

**Figure 1:**
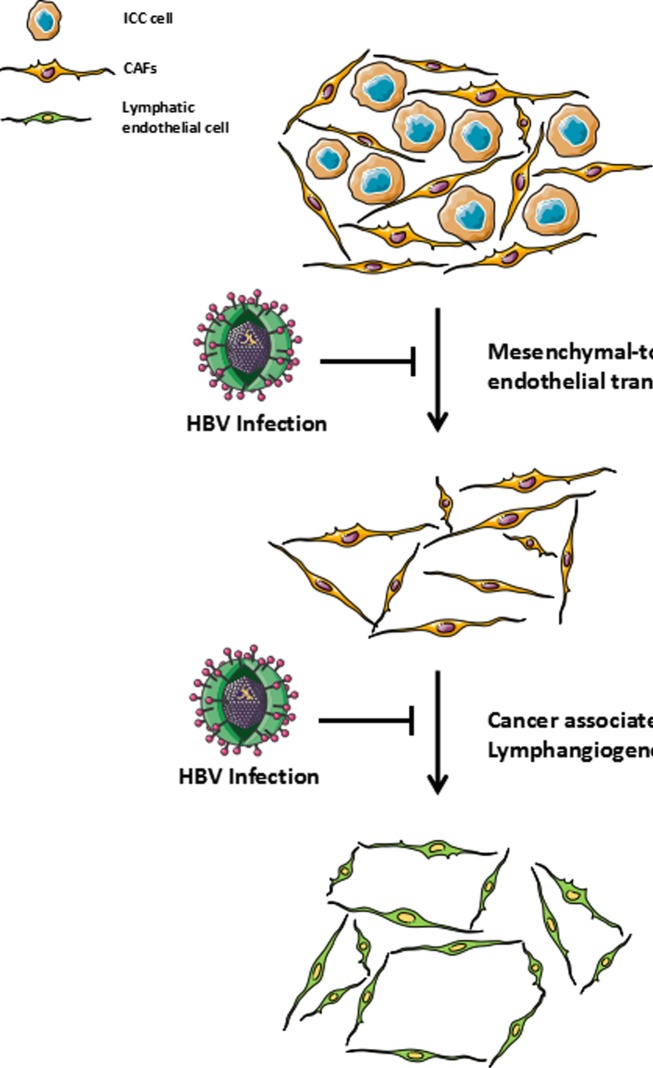
Role of HBV infection in cancer-associated lymphangiogenesis / HBV infection could be involved in the suppression of cancer-associated fibroblasts to adopt a lymphatic endothelial morphology and function (mesenchymal-to-lymphatic endothelial transition) which has resulted in the low extent of cancer-associated lymphangiogenesis and led to a relatively low incidence of lymph node metastasis in HBV-associated ICC

Most of relevant studies reported that lymph node metastasis was significantly reduced in HBV-associated ICC patients compared with those without HBV infection. However, the publications could not provide any pathological evidence of this phenomenon and there was no further explanation. Gurzu et al. [45] performed immunohistochemical staining on 15 Kaposi sarcoma specimens and found that human herpesvirus 8-modified pluripotent mesenchymal cells most likely transformed into vascular endothelial cells. More recently, a publication from *Nature* concluded that cardiac fibroblasts could be transformed into vascular endothelial cells, thereby participating in neovascularization after cardiac injury to improve cardiac function [46]. Along with the recent discovery of the existence of activated fibroblasts (cancer-associated fibroblast, CAF) in ICC and its potential ability to promote the development of the tumor by stimulating the invasive genotype of malignant cells, CAFs (mesenchymal cells) in stromal areas of ICC may also adopt an endothelial morphology and function [47, 48]. Therefore, reduced incidence of lymph node metastasis observed in HBV-associated ICC patients could be a result of inhibited cancer-associated lymphangiogenesis via suppressing mesenchymal-to-lymphatic endothelial transition in ICC (Figure 1). However, there is no conclusive mechanism-based evidence of mesenchymal-to-lymphatic endothelial transition in ICC, which awaits our future researches.

Recently, an imaging study of 40 nodules from 32 ICC patients with cirrhosis, showed manifestations of nodules that resulted in a heterogeneous contrast enhancement pattern, of which 19 cases presented with arterial peripheral-rim enhancement, 16 cases presented with a progressive homogeneous contrast uptake within the three vascular phases, and 2 cases were not detected by CT-scan [49]. Interestingly, the nodules did not reveal any radiological features of HCC; only ICC showed a homogeneous wash-in during the arterial and portal phases and a wash-out during the delayed venous phase, indicating that HBV-associated ICC in cirrhotic patients display distinct vascular characteristics on CT-scan that could be applied to differentiate from HCC [49].

## SURVIVAL OUTCOMES

In recent years, several publications evaluated the correlation between the presence of HBV infection and the survival outcomes of patients with ICC (Table 1). The majority of these studies insisted that HBV infection may be a favorable prognostic factor in patients with ICC [39, 40, 50–56]. However, there are still a few studies that reported HBV infection has no impact on the survival outcome of patients with ICC after hepatic resection [5, 31, 43, 57]. Zhang et al. [50] suggested that ICC patients with both current and past HBV infection [seropositivity of HBsAg and hepatitis B core antibody (HBcAb)] had a significantly better prognosis than patients without a history of HBV infection and deduced that the activated immune responses by current or past HBV infection may enhance anti-tumor activity against ICC. Zhou et al. [51] suggested that inhibited tumor invasiveness prolongs the survival outcome of HBV-associated ICC patients, insisting that HBV-associated ICC should be differentiated from HBV-negative ICC on account of different clinicopathological features and survival outcome. A mathematical model for predicting survival duration (total accuracy, 92.3%; a positive predictive value, 93.8%) of ICC patients after hepatectomy conducted by Jiang et al. [52] included HBV infection as a positive factor, further bolstering the objective evidence for HBV infection as a favorable prognostic factor. Wu et al. [53] also elucidated that HBV infection is a favorable prognostic factor for ICC patients due to early detection of the unexpected detection of ICC during chronic liver disease follow-up. Less than one third of HBV-associated ICC patients were found to be at stage III and IV, whereas the majority of ICC patients without HBV infection were at stage III and IV upon diagnosis. Moreover, Liu et al. [55] observed this phenomenon from a different point of view and discovered that both HBV infection and vaccination prior to surgical treatment, combined with preoperative adjuvant chemotherapy, were found to be independent prognostic factors that improved survival outcomes of patients when undergoing hepatic resection for ICC. On the contrary, Tao et al. [28] described that 1-, 3-, and 5-year cumulative survival rates of HBsAg-positive ICC patients are significantly lower than HBV-negative ICC patients (27.3%, 0%, and 0% vs. 87.5%, 66.7%, and 50.0%, P < 0.001). More recently, Ahn et al. [5] analyzed the survival outcomes of 37 HBV-associated and 255 HBV-negative ICC patients who underwent hepatic resection, and demonstrated that their postoperative outcomes showed no significant difference, but more favorable tumor features were observed in HBV-associated ICC patients due to a relatively earlier diagnosis.

**Table 1:** A comprehensive review of survival outcomes in HBV-associated ICC

Study	Study region	Marker detection	Patients (n)	Overall survival (%)		Median survival	P value	Outcomes
				1-yr	5-yr			
Zhang (2010) [50] HBV-ICC Non-HBV-ICC	China	HBsAg/ HBcAb	29 10	78.9 46.7	35.7 NR	32 m	0.025	Favorable
Zhou (2011) [51] HBV-ICC Non-HBV-ICC	China	HBsAg	87 68	72.4 45.6	41.8 (3-yr) 20.5 (3-yr)	NR	0.003	Favorable
Wu (2013) [53] HBV-ICC Non-HBV-ICC	China	HBsAg/ HBcAb	97 41	42 24	15 0	NR	0.005	Favorable
Liu (2013) [55] HBV-ICC Non-HBV-ICC	China	HBsAg/ HBcAb	37 23	45.9 30.4	15 (3-yr) 0 (3-yr)	12 m 6 m	0.017	Favorable
Luo (2014) [38] HBV-ICC Non-HBV-ICC	China	HBsAg	608 725	NR NR	NR NR	19 m 12 m	< 0.001	Favorable
Wu (2015) [54] HBV-ICC	China	HBsAg/ HBcAb	85	60	13	25 m	NA	NA
Tao (2016) [28] HBV-ICC HBcAb-ICC Non-HBV-ICC	China	HBsAg/ HBcAb	39 (total)	27.3 62.5 87.5	0 0 50	6.9 (mean) 17.0 (mean) 33.0 (mean)	< 0.001	Unfavorable
Ahn (2016) [5] HBV-ICC Non-HBV-ICC	Korea	HBsAg	37 255	NR	NR	NR	NS	No difference
Jeong [unpublished] HBV-ICC Non-HBV-ICC	China	HBsAg	56 64	72.2 50.0	46.5 (3-yr) 18.0 (3-yr)	21 m 11 m	0.001	Favorable

HBsAg: hepatitis B surface antigen, HBcAb: hepatitis B core antibody, NR: not reported, HBV-ICC: hepatitis B virus-associated intrahepatic cholangiocarcinoma, NA: not applicable, NS: not significant.

## PREOPERATIVE EVALUATION AND TREATMENT

To date, previous publications introduced curative ICC treatment heterogeneously, and all patients with ICC have been grouped into one category [58, 59]. Previous extrapolations from clinical trials associated with the treatment of ICC has been reported, providing abundant evidence for researchers to consider a clinical practice criteria from a pragmatic perspective, but the findings are still too various and limited to establish a unified standard therapy, especially in patients with HBV-associated ICC. According to one of the major hallmarks of ICC, which is frequent metastasis, several surgeons suggested a staging laparoscopy to be performed before or during the surgical operation. A previous publication reported that 6/22 ICC patients (27%) with undetected intrahepatic or peritoneal metastasis were found to have metastasis when laparoscopy was performed [63]. However, currently there is insufficient evidence to recommend routine staging laparoscopy as an indispensable inspection for patients with HBV-associated ICC.

In recent years, prophylactic lymph node dissection has been introduced as a favorable prognostic factor for patients with ICC [66, 67]. Murakami et al. [68] documented the first 5-year survival of a periductal-infiltrating ICC patient with para-aortic nodal metastasis after a wide dissection, including regional and para-aortic nodals, in combination with a hemi-hepatectomy was performed. In addition, one of our colleagues recently found that there was no difference in the survival outcomes between patients with para-aortic nodal metastasis and patients with only regional lymph node metastasis, suggesting that micrometastases of para-aortic nodes may also be associated with the prognosis of patients with ICC [69]. To date, there is no information considering whether preventive wide nodal dissection could be a benign factor for HBV-associated ICC. Therefore, prospective controlled investigations evaluating the efficacy of preventive lymphadenectomy in HBV-associated ICC patients are needed.

To date, in order to allocate organs to patients with the optimal chance of long-term survival, it is considered impractical to perform liver transplants (LT) on hepatic cancer with a 5-year survival rate of less 50%, leading to the current viewpoint that LT for ICC is unacceptable [70]. However, the guidelines from the International Liver Cancer Association recommend a thorough investigation of the standardized candidate criteria for treating ICC patients with LT in combination with neoadjuvant chemotherapy [71]. Our previous meta-analysis also revealed that neoadjuvant chemotherapy or radiotherapy followed by LT may be an alternative option in order to achieve a favorable prognosis for patients with unresectable ICC [72]. Liu et al. [55] reported that 18 of 81 patients who underwent hepatic resection in combination with adjuvant chemotherapy [transcatheter hepatic arterial chemoembolization (TACE) and systemic venous anti-neoplastic therapy] obtained a significantly prolonged 1-year and 3-year cumulative overall survival rates compared to patients who did not receive any adjuvant chemotherapy (1-year: 83.8%, 3-year: 33.3% vs. 1-year: 31.7%, 3-year: 4.1%; P = 0.015), emphasizing the indispensable role of neoadjuvant chemotherapy or radiotherapy in the treatment of ICC. As described above, HBV-associated ICC demonstrated a significantly lower tumor recurrence and more prolonged long-term survival. Therefore, it is strongly recommended that HBV-associated ICC needs to be treated with neoadjuvant chemoradiotherapy followed by LT and adjuvant chemotherapy.
